# Electromechanical efficiency index of skeletal muscle and its applicability: a systematic review

**DOI:** 10.3389/fbioe.2024.1398047

**Published:** 2024-05-09

**Authors:** Gasper Turnsek, Armin Huso Paravlic

**Affiliations:** ^1^ Institute of Kinesiology, Faculty of Sport, University of Ljubljana, Ljubljana, Slovenia; ^2^ Science and Research Centre Koper, Institute for Kinesiology Research, Koper, Slovenia; ^3^ Faculty of Sports Studies, Masaryk University, Brno, Czechia

**Keywords:** EME index, muscle function, tensiomyography, electromyography, rehabilitation, athletic performance

## Abstract

**Introduction:** The electromechanical efficiency of skeletal muscle represents the dissociation between electrical and mechanical events within a muscle. It has been widely studied, with varying methods for its measurement and calculation. For this reason, the purpose of this literature review was to integrate the available research to date and provide more insights about this measure.

**Methods:** A systematic search of the literature was performed across three online databases: PubMed, ScienceDirect, and SPORTDiscus. This yielded 1284 reports, of which 10 met the inclusion criteria. Included studies have used different methods to measure the electromechanical efficiency (EME) index, including electromyography (EMG), mechanomyography and tensiomyography (TMG).

**Results:** The EME index was used to assess muscle conditions such as muscle atrophy, pain syndromes, or to monitor rehabilitation in patients with knee problems, fatigue and the effects of exercise and rehabilitation. TMG has been shown to be one of the most reliable methods to obtain the EME index, but its use precludes obtaining the index during voluntary muscle contractions.

**Conclusion:** Standardizing the EME index is crucial for its diverse applications in clinical, sport, and rehabilitation contexts. Future research should prioritize standardization of measurement protocols for establishing the most repeatable, and reliable approach that can be used for inter-individual comparisons or for assessing an individual for multiple times over a longer period.

**Systematic Review Registration:** https://www.crd.york.ac.uk/prospero/display_record.php?ID=CRD42023440333 Identifier: CRD42023440333.

## 1 Introduction

Muscle contraction occurs as a result of electromechanical coupling, which determines the shortening of sarcomeres and hence of entire muscle fibers, while the simultaneous contraction of thousands of muscle fibers causes the muscle to shorten in the longitudinal direction (Valencic et al., 2001; Macgregor et al., 2018). Mechanical muscle function can be quantified using a relatively new non-invasive technique, mechanomyography (MMG), that evolved from sensing muscle vibrations into a sophisticated tool that can capture changes in the geometry of muscle fibers during contractions, specifically lateral oscillations generateded by active muscle, and its own resonance frequency (Ibitoye et al., 2014). These oscilations are independent of the electrical activity of the motor units as measured by electromyography (EMG) (Beck et al., 2005). Specifically, the MMG amplitude is thought to reflect motor unit recruitment, whereas the EMG amplitude reflects both motor unit recruitment and the degree of motor unit integration (Farina and Enoka, 2023). Recent advances in sensor technology and signal processing have improved the capabilities of MMG, giving it insight into muscle mechanics and motor unit activation patterns. Specifically, the MMG amplitude is thought to reflect motor unit recruitment, whereas the EMG amplitude reflects both motor unit recruitment and the degree of motor unit integration (Ebersole and Malek, 2008).

The electromechanical efficiency of skeletal muscle represents the dissociation between electrical and mechanical events of muscle function (i.e., electromechanical coupling), which can be captured by examining the changes in the ratio between MMG and EMG amplitudes, thus creating an electromechanical efficiency (EME) index (Barry et al., 1990). The importance of quantitative insight into the electromechanical function of the vastus medialis muscle during ergometer cycling was highlighted in the study Oka and colleagues (Oka et al., 2014). Authors combined EMG and MMG assessment to objectively quantify muscle performance during dynamic exercise. Several authors have emphasized this approach as simple and desirable in sports and rehabilitation due to different advantages, such as simultaneous assessment of muscle activation and mechanical properties (Beck et al., 2005; Anders et al., 2019; Fukuhara et al., 2021). This provides a comprehensive insight into muscle function and its non-invasive nature, making it practical for use in a variety of settings, from clinical rehabilitation to athletic training (Beck et al., 2005; Anders et al., 2019; Fukuhara et al., 2021).

The EME analysis can provide important information about the status of various muscles including facial and limb muscles (Ioi et al., 2006). It has been used in clinical settings to assess muscle atrophy after disease (Berg et al., 1997), or as a potential indicator of patellofemoral pain syndrome and to assess muscle adaptation after exercise (Ebersole and Malek, 2008). A quite recently, EME index has also been used to assess lower limbs muscle function changes following total knee replacement in end-stage osteoarthritis patients (Paravlic et al., 2020). The EME index can discriminate concentric and eccentric contraction, as well as changes in intrinsic contractile muscle properties after experimentally induced muscle pain (Madeleine and Arendt-Nielsen, 2005). The later findings arguing the importance of EME index, as EMG alone was not being sensitive enough to detect those changes as MMG did during simultaneous measurements (Madeleine and Arendt-Nielsen, 2005). The EME index showed to be useful tool to discriminate between apparently healthy and subjects diagnosed with muscular dystrophy, as reduced EME index has been found in symptomatic compared to healthy subjects (Barry et al., 1990). The age-related muscular dysfunction was investigated using the EME index on patients with chronic low back pain to understand the electrical and mechanical aspects of the pain induced (Sakai et al., 2019). A comparison of EME between vastus medialis and vastus lateralis muscles was performed in a study conducted by (Ebersole and Malek, 2008). EME measurements offer a distinctive perspective on the impact of fatigue on skeletal muscle contractile properties, encompassing changes in intrinsic electric and mechanical components. EME might be valuable in evaluating clinically significant asymmetries in vastus medialis and vastus lateralis muscle function among individuals with knee injuries. The utility of the above has been applied to evaluation of plyometric training effect on the gastrocnemius muscle electromechanical properties (Zubac et al., 2019). Authors reported improvement of EME index in gastrocnemius muscle following 9 weeks of plyometric training in older adults.

The aforementioned studies substantially varied in methods used to evaluate the EME index. The most common methods of MMG signal acquisition refer to acoustomyography using microphones (Jaskólski et al., 2007), vibromyography using piezoelectric accelerometers (Madeleine and Arendt-Nielsen, 2005; Ebersole and Malek, 2008; Sakai et al., 2019); and measurement of perceived skin over muscle movements by detecting changes in the magnetic field (Ioi et al., 2006). A novel method of obtaining the EME index is using tensiomyography (TMG), designed to assess the evoked contraction of individual superficial skeletal muscles (Paravlić et al., 2017; Paravlic et al., 2020). TMG is essentially an MMG method where a TMG sensor is used to detect radial displacement of muscle belly. In study of Paravlic and colleagues, it was used simultaneously with EMG signal (M-wave) to obtain EME index. From the TMG response, the peak-to-peak amplitude of muscle radial displacement (Dm, measured in millimetres [mm]) and the peak-to-peak amplitude of the M-wave (Mptp) are analyzed. It can be said that TMG uses the same principles as mechanomyography, but is designed to work only when the muscle contraction is electrically elicited and uses a unique sensor to detect radial muscle displacement (Valencic et al., 2001). The EME index obtained using tensiomyography was calculated as the ratio of Dm to Mptp (Paravlić et al., 2017).

By overview of the published literature, we found that different methods were used to measure and calculate the EME index. These includes EMG, MMG, and TMG. Moreover, the EME index was used for different purposes and among different populations. For this reason, the purpose of this literature review was to integrate the available research to date and to provide the answers on the following questions: a) What methods have been used so far to measure and calculate the EME index; b) Evaluate the strengths and weaknesses of these methods; c) Suggest guidelines for future research in this field.

## 2 Methods

The review was carried out in accordance with PRISMA 2020 guidelines (Page et al., 2021). The protocol for present study was prospectively registered on PROSPERO online registry (ID: CRD42023440333).

### 2.1 Exploratory search strategy

The literature review was carried out by searching PubMed, ScienceDirect, and SPORTDiscus databases from 17 to 27 July 2023. No restriction on the year of publication or language was applied. In all databases, the following keywords or phrases were used: “Electromechanical efficiency,” “Electromechanical efficiency index,” “EME,” “EME index,” “Muscle,” “Skeletal muscle.” The Boolean “OR” and “AND” where used where possible.

### 2.2 Eligibility criterion for selecting a study

The inclusion criteria were formulated based on the PICO approach, which covers population (P), intervention (I), comparison (C) and outcome (O) (Page et al., 2021): P—men or women of all ages; I—any intervention that used an electromechanical index for neuromuscular status assessment regardless of the method of measurement; C—the case of original research designs with a control group, the control group would be a stand-alone intervention, and in the case of cross-sectional studies, the comparison would look at different individuals (older *versus* younger, left *versus* right limb, non-affected *versus* affected limb, etc.), and; O—electromechanical efficiency of skeletal muscles.

Studies were excluded according to the following criteria: 1) studies that did not investigate the electromechanical efficiency of skeletal muscle and, 2) not meeting inclusion criteria mentioned before.

### 2.3 Screening strategy

The screening was performed by the first author (GT). After the first screening by GT, the screening was performed by the second author (AP), and based on a compromise between the two authors, the study was included or excluded from the review. As the original studies did not include randomized controlled trials with an experimental design, the methodological quality of the studies was assessed using the Quality Assessment Tool for Observational Cohort and Cross-Sectional Studies developed by National Heart, Lung, and Blood Institute, National Institute of Health (National Heart Lung, and Blood Institute, 2021).

### 2.4 Data extraction

The information about study design, population (sample type, size and age), perceived variable—EME (mode of integration and which muscles/locations it was performed on) and additional information (tools used in the study and its purpose) were extracted from the original studies included in present review.

### 2.5 Methodological quality assessment

The Quality Assessment Tool for Observational Cohort and Cross-Sectional Studies (National Heart Lung, and Blood Institute, 2021) was used to assess the methodological strength and risk of bias of the 9 studies included in the review. The methodological assessment of the studies was performed independently by both authors. The NIH Quality Assessment Tool consists of a checklist of 14 questions designed to assess the internal validity (potential risk of selection, information, or measurement bias) of cross-sectional and cohort studies. The criteria were answered “yes,” “no,” or other (not specified; not applicable; not reported). The total score would be the number of affirmative responses. For the qualitative assessment of the final score, scores higher than 12 were considered good, those lower than 9 were considered weak, and those falling in the range 9–12 represented moderate-quality studies. All included studies were rated as good, fair, or poor quality on the basis of a rating sheet with quality assessment instructions.

## 3 Results

### 3.1 Basic characteristics of the included studies

**FIGURE 1:**
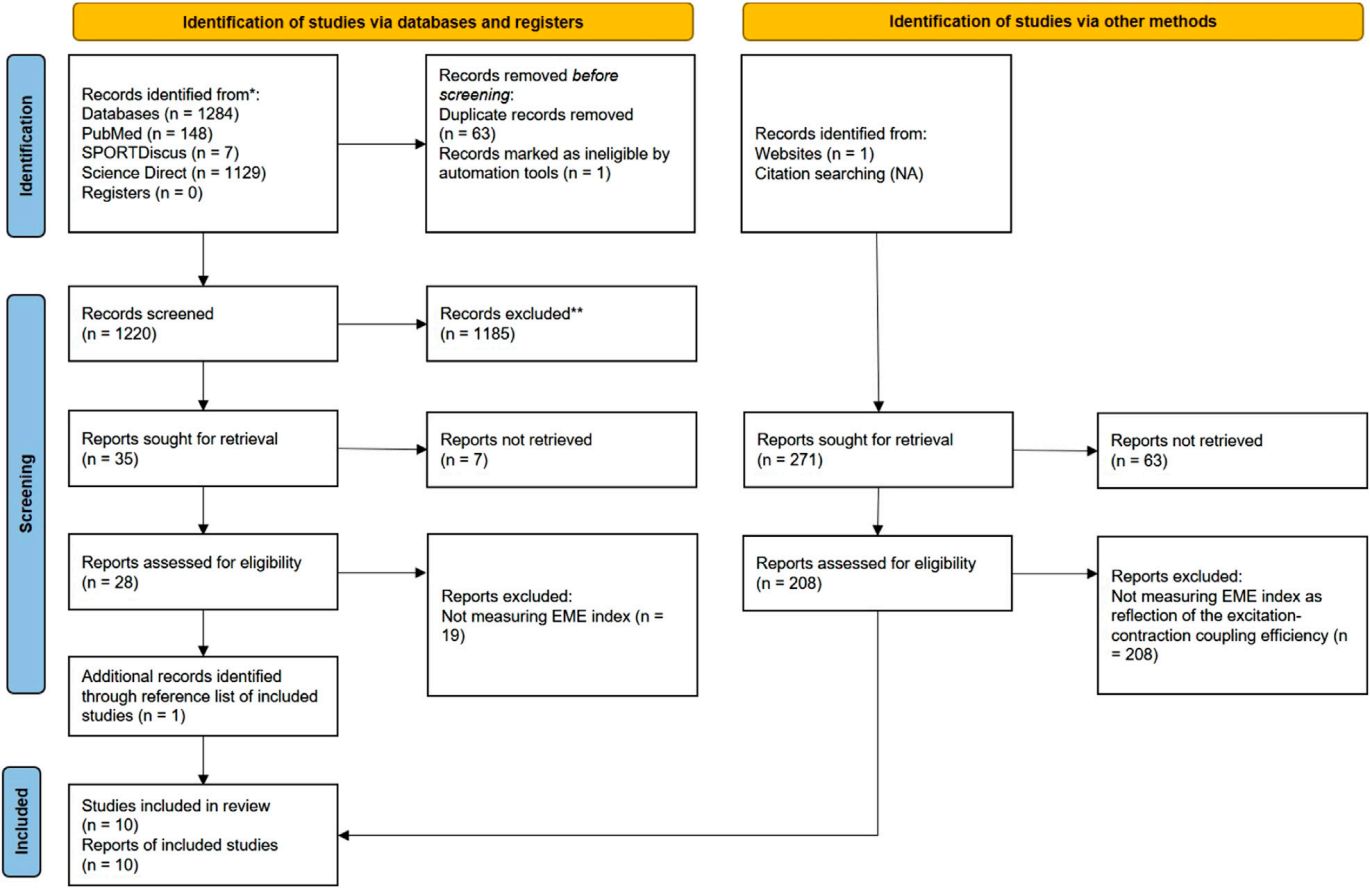
PRISMA flow diagram of the literature selection process.

The process of searching and selecting articles is presented in Figure 1. In total, ten articles met the inclusion criteria. The basic characteristics of the studies and subjects are presented in Table 1. The total number of subjects in all studies was 267, and their mean age was 38.69 ± 4.02 years. In all studies, the main inclusion criterion was that the EME index reflects the excitation-contraction process in active skeletal muscle. In most studies, the EME index was obtained by EMG and MMG analysis. An exception was the use of the new TMG technology (Zubac et al., 2019; Paravlic et al., 2020). The EME index obtained using TMG technology was shown to be highly reliable in the study by Paravlić et al. (2017). The EME index was used over large superficial muscles in all included studies.

**TABLE 1:** Characteristics of the included studies.

Authors	Type of study	Sample characteristics (type of sample/Size/Age)		Search variable (EME)		Additional		
		Experimental group	Control group	Method of integration	Muscles/Location	Tools used	Purpose	Findings
Paravlic et al. (2020)	Cross-sectional study	14 M and 12 W before and after TKA/61.12 ± 5.34	—	The ratio of Dm to M_ptp_ (in mm/mV)	Gastrocnemius medialis	TMG, EMG, Electrical Stimulator, Isometric dynamometer	Investigate muscle-specific changes in lower extremities, physical function of patients and EME muscle gastrocnemius medialis in early post-TKA period using TMG	Muscle contractile properties gradually improve during the rehabilitation period after TKA. This also increases the EME index, which indicates a better conversion of electrical activation into mechanical work. Therefore, they clarify that the index allows an objective assessment of muscle performance and can serve as an important indicator when planning a rehabilitation program
Sakai et al. (2019)	Cross-sectional study	55 W and 61 M with chronic low back pain/73.17 ± 6.45	—	MMG-RMS to EMG-RMS ratio between each lumbar deflection and extension	Multifidus, Erector spinae	MMG, EMG, MRI, X-ray	Evaluate electrophysiological activation of the multifidus and erector spinae muscles in elderly patients with chronic low back pain	Elderly patients with chronic lower back pain showed decreased lumbar multifidus muscle index and erector spinae compared to healthy control groups. This indicates a lower ability to convert electrical activation of muscles into a force generated in patients with chronic lower back pain
Zubac et al. (2019)	RCT	Persons of daily activity centres in Primorska/23/66.7 ± 5.2	—	The ratio of Dm to M_ptp_ (in mm/mV)	Gastrocnemius medialis	TMG, EMG, Electrical stimulator, Force plate	To study the effects of 8 weeks of controlled plyometric exercise on jumping efficiency, contractile performance and inflammatory response in the elderly	Research findings showed that plyometric exercise affected the EME muscle index in older people. An increase in EME may be associated with an improvement in muscle fiber coordination and activation
Grosprêtre et al. (2018)	Cross-sectional study	11 M, practicing Parkout/21 ± 3	10 m recreationally involved in sports/23 ± 3	The ratio of the maximum muscle displacement triggered at M_max_ (Σ M_max_)	Soleus, Gastrocnemius medialis	MMG, EMG, Dynamometer	To assess the impact of many years of explosive strength training, which can form the neuromuscular profile of athletes	The EME index has proven to be a key factor that distinguishes Parkout athletes from other athletes. These showed significantly higher EME indices, which indicates their ability to generate greater mechanical power per unit of electrical activation
Paravlić et al. (2017)	Cross-sectional study	10 M and 8 W/30.3 ± 10.3	—	The ratio of Dm to M_ptp_ (in mm/mV)	Soleus	TMG, EMG, Electrical stimulator	Examine the reliability of the EME during the day, during days and between assessors	High reliability of the electromechanical efficiency assessment by means of the ratio of TMG (Dm) and M-wave amplitudes was established. Repeated measurements led to similar results, which confirms the stability of this method and the reliability of the measurements obtained
Ebersole & Malek, (2008)	Quasi experiment	Healthy M/10/23 ± 1.2	—	The ratio of normalized MMG amplitude to normalized EMG amplitude (each iteration separately)	Vastus medialis, Vastus lateralis	EMG, Isokinetic, MMG	Investigate the relationship between muscle fatigue and electromechanical efficiency	It was found that fatigue had a significant impact on the electromechanical efficiency of both muscles. After loading, EME was reduced by 58% and 66%, respectively, indicating a reduced ability of muscles to convert electrical activation into mechanical work
Jaskólski et al. (2007)	Cross-sectional study	18 M/23 ± 1.3	—	The ratio of MMG RMS/EMG RMS [mV/µV]	Biceps brachii, Triceps brachii	EMG, Isokinetic, MMG	Investigate the impact of eccentric contractions on muscle biceps and triceps brachii during MVC bends of the elbow joint using electrical (EMG) and mechanomyographic activities (MMG)	Electromechanical efficiency played a key role in muscle response after eccentric exercise, where a similar response from both agonistic and antagonistic muscles was found. The high EME index was associated with a greater ability of muscles to convert electrical muscle activation into mechanical strength
Ioi et al. (2006)	Cohort study	16 M/25.6 ± 2.3	—	Mean corrected value (ARV) for MMG (mm)/ARV for EMG (x10^−^ ^2^ mV)	Masseter	EMG, MMG, Bite force sensor	Study MMG validation to assess Masseter muscle fatigue (masticatory muscle)	The results showed that jaw muscle fatigue was associated with reduced EME. Reduced EME indicates a reduced ability of muscles to convert electrical activation into mechanical work, which in turn can affect jaw functionality, such as reduced bite strength and difficulty performing certain chewing tasks
Madeleine et al. (2001)	RCT	Healthy M/13/24.4 ± 1.1	—	The ratio of MMG-RMS to EMG-RMS	First Dorsal interosseous	EMG, MMG, Potentiometer	Systematically investigate whether complementary knowledge can be gained from images of electromyography (EMG) and mechanomyography (MMG) signals	The study found that the type of muscle contraction, the rate of contraction and angular velocity affected the EME index. Eccentric contraction results in a higher EME index than in concentric muscle contractions
Barry et al. (1990)	RCT	Children with neuromuscular disease/16/7–16	Children without neuromuscular disease/11/7–16	The ratio of AMG to EMG	Biceps brachii	AMG, EMG, Force Transducer, microphone	Understand the electromechanical efficiency of muscle activity and its variations in normal muscle function compared to muscle affected by myopathy or muscular dystrophy in children	The results showed that the ratio between AMG and EMG, especially when analyzed with a linear discriminant function, holds promise as a diagnostic tool for distinguishing between normal and diseased muscles in the pediatric population. Increased ratios in patients indicate changes in electromechanical efficiency associated with childhood muscle diseases

Legend: AMG, acoustic myography; RCT, randomised control trial; EMG, electromyography; MMG, mechanomyography; MVC, maximum voluntary contraction; RMS, root mean square; TMG, tensiomyography; Dm, maximum; TMG, amplitude; Mptp, M-wave peak-to-peak; TKA, total knee arthroplasty; MRI, magnetic resonance imaging; X-ray, high-energy electromagnetic radiation.

### 3.2 Methodological quality assessment

Both authors (GT and AP) blinded to each other’s results, screened the full text against the NLHBI criteria and rated methodological quality independently. The median NIH score was 9.9 (SD:1.4), with values ranging from 8 to 12, suggesting that the included studies were generally of fair quality (Table 2).

**TABLE 2:** The methodological quality of included studies assessed by the quality assessment tool for observational cohort and cross-sectional studies.

Reference	Criteria 1	Criteria 2	Criteria 3	Criteria 4	Criteria 5	Criteria 6	Criteria 7	Criteria 8	Criteria 9	Criteria 10	Criteria 11	Criteria 12	Criteria 13	Criteria 14
Paravlic et al. (2020)	YES	YES	NA	YES	YES	YES	YES	YES	YES	YES	YES	NR	NR	YES
Sakai et al. (2019)	YES	YES	CD	YES	YES	YES	CD	YES	YES	CD	YES	CD	YES	YES
Zubac et al. (2019)	YES	YES	CD	YES	YES	YES	YES	YES	YES	YES	YES	CD	YES	YES
Grosprêtre et al. (2018)	YES	YES	CD	YES	YES	YES	YES	YES	NR	YES	CD	CD	YES	YES
Paravlić et al. (2017)	YES	YES	NA	YES	YES	YES	NA	NA	YES	YES	YES	NA	NA	NA
Ebersole & Malek, (2008)	YES	YES	NR	YES	YES	YES	YES	YES	YES	YES	YES	NA	NA	YES
Jaskólski et al. (2007)	YES	YES	YES	YES	YES	YES	CD	CD	YES	YES	YES	CD	CD	CD
Ioi et al. (2006)	YES	YES	NA	YES	YES	YES	YES	YES	NR	YES	YES	NR	YES	YES
Madeleine et al. (2001)	YES	YES	NA	YES	YES	YES	NA	YES	YES	NA	YES	NA	NA	YES
Barry et al. (1990)	YES	YES	YES	YES	NR	YES	YES	NR	YES	NR	YES	NR	NR	NR

NA, not applicable; NR, not reported; CD, cannot determine; Criteria 1—Was the research question or objective in this paper clearly stated?; Criteria 2—Was the study population clearly specified and defined?; Criteria 3—Was the participation rate of eligible persons at least 50%?; Criteria 4—Were all the subjects selected or recruited from the same or similar populations (including the same time period)? Were inclusion and exclusion criteria for being in the study prespecified and applied uniformly to all participants?; Criteria 5—Was a sample size justification, power description, or variance and effect estimates provided?; Criteria 6—For the analyses in this paper, were the exposure(s) of interest measured prior to the outcome(s) being measured?; Criteria 7—Was the timeframe sufficient so that one could reasonably expect to see an association between exposure and outcome if it existed?; “Criteria 8—For exposures that can vary in amount or level, did the study examine different levels of the exposure as related to the outcome (e.g., categories of exposure, or exposure measured as continuous variable)?; Criteria 9—Were the exposure measures (independent variables) clearly defined, valid, reliable, and implemented consistently across all study participants?; Criteria 10—Was the exposure(s) assessed more than once over time?; Criteria 11—Were the outcome measures (dependent variables) clearly defined, valid, reliable, and implemented consistently across all study participants?; Criteria 12—Were the outcome assessors blinded to the exposure status of participants?; Criteria 13—Was loss to follow-up after baseline 20% or less?; Criteria 14—Were key potential confounding variables measured and adjusted statistically for their impact on the relationship between exposure(s) and outcome(s)?

### 3.3 Detailed presentation of the results of the included studies

The experimental research to gain complementary knowledge from EMG and MMG signal recordings was conducted by Madeleine and colleagues in 2001 (Madeleine et al., 2001). Grosprêtre and colleagues compared EME index of triceps surae muscles between power trained athletes engaged in parkour and untrained individuals. Authors found a higher electromechanical efficiency in parkour athletes confirming the greater excitation-contraction coupling efficiency in power trained athletes then non-trained individuals (Grosprêtre et al., 2018). The reliability of the EME obtained by TMG was presented in study of (Paravlić et al., 2017). EME was used to compare muscles before and after muscle fatigue in two studies (Ioi et al., 2006; Ebersole and Malek, 2008), and for comparison between healthy muscles and affected muscles due to chronic knee osteoarthritis (Paravlic et al., 2020) or lower back pain (Sakai et al., 2019). Finally, Zubac and colleagues used EME index to evaluate the effects of plyometric training on medial gastrocnemii muscle in older adults, whereas Jaskólski and colleagues (Jaskólski et al., 2007) used EME index to investigate the effects of acute eccentric training on agonist and antagonist muscles during the elbow flexion movement.

## 4 Discussion

The aim of this systematic literature review was to investigate various aspects of the EME index, including its utility in assessing muscle conditions, muscle atrophy, pain syndromes, and the effects of exercise and rehabilitation. Additionally, it aimed to provide guidelines for further research and use of the EME index. Ten relevant studies were identified that employed the EME index for diverse purposes and utilized different methods to capture electro-mechanical signals of skeletal muscle. Overall, the EME index appears to offer detailed insights into muscle function and holds potential clinical relevance, as discussed in the following sections.

### 4.1 Methodology for estimating the EME index

The EME index is a measure of muscle function derived from the assessment of electrical and mechanical muscle actions. The most common calculation technique is the ratio of MMG RMS (root mean square) to EMG RMS [mV/uV] (Madeleine et al., 2001; Jaskólski et al., 2007; Sakai et al., 2019). The Ebersole & Malek, (2008) used the relationship between the normalized MMG amplitude and the normalized EMG amplitude (each repetition separately), by incorporating linear, quadratic and cubic polynomial regression models. A specific calculation of the EME index appears in the study of Ioi and colleagues, where the calculation is made from average rectified value for MMG and EMG values in order to determine how much muscle action potential is converted into muscle contraction. In other studies used a new MMG technique called TMG, where the EME index was calculated using the parameter extracted from the TMG (Dm) and the M-waves (Mptp) to form the EME index (Dm/Mptp) (Paravlić et al., 2017; Zubac et al., 2019; Paravlic et al., 2020). A similar extraction strategy was used in the study of Grosprêtre et al. (2018) where the EME index was obtained from the ratio of the average muscle twitches measured by MMG obtained at the maximum M-wave (Mmax). Given that both methods used in later studies calculate the EME index from electrically elicited muscle contraction in isometric conditions, it can be said that these two methods are the most similar, differing primarily in the assessment of the maximum mechanical response of the muscle. Common to all studies is the introduction of the EME index, representing the ratio of the mechanical response normalized to the electrical muscle activity. However, the question arises as to which method of EME index derivation is the most reliable, a factor dependent on how the signals are acquired and processed. The measurement of the mechanical response of muscles varies between studies, utilizing accelerometers, piezoelectric contact sensors, condenser microphones, laser distance sensors, etc., but the reliability of these methods poses a challenge (Ibitoye et al., 2014). For instance, a considerable amount of the variation in the reliability among the above-mentioned methods might be explained by the initial setting of the measurements, which differs between methods studied. This includes factors such as the type of muscle contraction studied (isometric vs dynamic; voluntary or electrically elicited). For example, Al-Zahrani and colleagues (2009) employed the isometric contractions, where the reliability of the MMG signal in the assessment of quadriceps fatigue was investigated and found to be high (ICC = 0.79–0.83) and low (ICC = 0.43–0.66) for intra-day and inter-day assessments, respectively. Paravlić and colleagues (2017) summarised the findings of other studies where different muscles are studied but under the same conditions (isometric contraction) and reported a very high coefficient of variance among studies. Authors also found the highest reliability measured by acoustomyography (excluding the TMG technique) that is achieved at the lowest levels of muscle activity. During the analysis of dynamic muscle function, which is of greater importance in clinical applications numerous factors affecting MMG have been identified (Stokes, 1993; Herda et al., 2008). These include changes in the length of the muscle and consequently torque, the ambient temperature, and the thickness of the tissue overlying the muscle being studied (Herda et al., 2008; Ibitoye et al., 2014). MMG obtained by means of a capturing radial muscle displacement triggered by an electrical stimulation pulse has been shown to be much more reliable compared to voluntary contractions (Ibitoye et al., 2014). These parameters seem to be reliable physiological parameters of the measured muscles that highly correlate with torque measurements and have also been used to assess muscle fatigue, stiffness and endurance, respectively (Uwamahoro et al., 2021).

A TMG represents a specific MMG technique that captures radial displacement of muscle belly after electrically elicited muscle contractions. The results of a quantitative summary of individual reliability studies confirm high to excellent relative reliability for all the basic TMG parameters including (muscle displacement (ICC, Dm = 0.98), time of contraction (Tc = 0.95) and time of delay (Td = 0.91)) (Lohr et al., 2019). Thus, Dm was used to calculate the EME index when utilizing the TMG method. Paravlic and colleagues showed very high reliability with an average ICC of 0.88 for Dm, 0.90 for Mptp and 0.92 for the EME index (Paravlić et al., 2017). This was also the first study to demonstrate the high reliability of the EME index obtained using the TMG and M-wave techniques. It can be said that the most appropriate and reliable method of obtaining the EME index is by TMG technique. It must be stressed that TMG operates during isometric muscle conditions and electrically induced muscle contraction with submaximal electrical stimuli. Further studies aimed at investigating the discriminative validity of EME index are warranted.

### 4.2 The EME index’s applied relevance

The EME index has been shown to have clinical relevance (Ebersole and Malek, 2008; Paravlic et al., 2020). The authors reported that EME provides insight into the impact of muscle fatigue on skeletal muscle function and is a useful tool to assess and quantify clinically relevant asymmetries in vastus medialis and vastus lateralis muscle function in patients with knee problems (Ebersole and Malek, 2008). Based on these findings, the EME index can be used as a screening tool to monitor rehabilitation protocols, in particular patellofemoral syndrome, or to improve exercise control and muscle function. Furthermore, Paravlic and colleagues assessed the EME index of gastrocnemii medialis muscle following the total knee arthroplasty that decreased for 38% (Paravlic et al., 2020). Orizio and colleagues (Orizio et al., 1997) investigated difference in muscle function between healthy and affected muscles in patients with cerebral palsy and muscular dystrophy. Observed differences in reduced electromechanical coupling in affected muscles were prescribed to reduction in type II muscle fiber content and overall number of efficient motor units in affected subjects (Orizio et al., 1997). Similarly, Barry and colleagues, suggested that the reduction in the EME index in adolescents with various neuromuscular disorders may be due to atrophy of muscle fibers which, although generating electrical activity, have very little mechanical activity (Barry et al., 1990). By providing objective measurements of muscle function and quality, clinicians can assess asymmetries, monitor progress, and tailor interventions to individual patient needs to improve outcomes across different clinical settings.

Given that the EME index has been shown to be clinically relevant, it would be reasonable to translate its usefulness into sports diagnostic practice. It has been shown to be a valid tool for the diagnosis of muscle fatigue, and this was confirmed in a study Ioi et al. (2006) where the mean EME values after fatigue were lower than those of the pre-fatigue trials at all levels expressed as a percentage of maximal voluntary contraction, namely, for the masseter muscle. In the study Kapadia, (2022) the authors report similar findings, namely, that the EME index has the potential to help characterise the influence of fatigue on force production. These factors cannot be accurately determined from EMG and MMG data alone, as a decrease in the index may be explained by impaired coupling of actin and myosin during muscle contraction, as a result of the accumulation of metabolic by-products, namely, hydrogen ions and diprotonated phosphate (Layzer, 1990). The sport-diagnostic relevance of the EME index has been demonstrated in a study by Jaskólski et al. (2007) who observed a reduction in EME values following 25 eccentric contractions at 50% MVC compared to pre-protocol. The authors reported that EMG and MMG recordings were differently altered immediately after and 120 h after the eccentric protocol, suggesting that several factors, including a) reduced rate of calcium release from the sarcoplasmic reticulum (acute effect); b) altered motor control (chronic effect); and c) increased muscle stiffness (chronic effect), mediated these results. These findings suggest that simultaneous measurements of electromechanical coupling may provide more insights into the mechanisms driving changes in neuromuscular function following the fatiguing protocols, than either parameter alone.

Despite all the sports-diagnostic advantages brought about by the introduction of the EME index, we cannot avoid the fact that the latter can be the simplest tool for evaluating the training cycle, which is the foundation of any successful training process. A 10-week plyometric training program has been shown to increase the EME index by 22.9% compared to a control group that continue with their habitual physical activity (Zubac et al., 2019). Therefore, we can conclude that the EME index can serve as useful tool for evaluating the electromechanical changes after physical training, as well as an indicator of improved jumping performance. Moreover, in the study conducted by Grosprêtre et al. (2018), EME index showed that parkour athletes had a significantly higher EME index compared to untrained athletes. Thus, suggesting that EME index may be used to distinguish more and less trained athletes.

### 4.3 Limitations of this review article

The findings of all the inclusion studies demonstrate the benefits offered by the introduction of an EME index, but some limitations should also be highlighted. One of these is certainly the number included studies. However, it is important to note that the introduction of an EME index is still a relatively new method with a limited body of research. For this reason, we believe that more studies are needed to investigate other emerging questions where EME index can serve its purpose in differentiating between several muscle groups, various exercise modalities and populations. The difficulties arising from crosstalk from adjacent muscles, which can affect the reliability of EMG and MMG signal capture, have been highlighted and this alone represents a major limitation for incorporating EME index into clinical practice. This could lead to misinterpretation of the results and limit the usefulness of the EME index. Further research should focus more on developing and testing methods to reduce the error of measurement in order to provide more reliable assessment. A limitation can also be seen in the limited generalisability of the results obtained. Many of the included studies focused on specific populations, such as athletes or people with specific health conditions. Despite all the results pointing in favour of the EME index, it would be reasonable to generalise the meaning in the sense that the EME index could be used as a measurement tool to determine muscle status in the general population. Also, one of the limitations is that included studies is extremely difficult to compare, as they use different approaches to obtain the EME index. For the latter reason, it would be useful to consider standardising the methods used to measure the EME index, as this would allow better comparability of results and facilitate the future research in this field.

## 5 Conclusion

The EME index is a promising parameter that provides a comprehensive and in-depth view of muscle function by describing the excitation-contraction process of skeletal muscle. The EME index is calculated by comparing the mechanical muscle response with its electrical potentials. The latter can be used to assess muscle conditions such as muscle atrophy, pain syndromes, or to monitor rehabilitation in patients with knee problems, fatigue and the effects of exercise and rehabilitation. Therefore, it can be said that the EME index has both clinical and sport-diagnostic relevance.

Various studies have used different methods to measure the EME index, including EMG, MMG, and TMG. TMG has been shown to be one of the most reliable methods to obtain the EME index, but its use precludes obtaining the index during voluntary muscle contractions.

Given the diversity of applicability and the different methods of capturing the EME index, which are presented in the paper, we expect advances in technology to obtain the EME index and thus improvements in psychometric properties. With standardized protocols, EME index can facilitate widespread use in clinical settings. In addition, ongoing research may uncover a new holistic insight into muscle function that helps with targeted clinical conditions.

Improved understanding and use of the EME Index is likely to lead to specific changes in clinical, sports and rehabilitation settings. Clinically, it could facilitate earlier detection and more targeted interventions for muscle dysfunction whereas in sports, it could be used for optimization of training regimes and injury prevention strategies. From arehabilitation point of view, it could help with personalised treatment plans and improved patient outcomes.

## Data Availability

The original contributions presented in the study are included in the article/Supplementary Material, further inquiries can be directed to the corresponding author.
